# Beyond Fair Labelling: Offence Differentiation in Criminal Law

**DOI:** 10.1093/ojls/gqac007

**Published:** 2022-05-28

**Authors:** Andrew Cornford

**Keywords:** fair labelling, offence differentiation, blame, blameworthiness

## Abstract

How should criminal conduct be divided among different offences? To date, this question has received only one serious answer: the fair labelling principle, which states that distinctions among offences should reflect distinctions in the nature and seriousness of the wrongdoing that they criminalise. This article argues that the fair labelling principle should not be the sole or main principle governing offence differentiation decisions. Its argument consists in three main claims. First, the only plausible foundation for the principle is a duty to ensure that the blame expressed through criminal conviction is allocated justly. Second, this duty cannot be absolute: if it were, the result would be an absurdly highly differentiated criminal law. Third, several other factors are relevant to how we should differentiate offences, and these will often count against the demands of just blaming. A complete normative account of offence differentiation must thus extend beyond fair labelling—or indeed, any single principle.

## 1. Introduction

Suppose we agree that a given behaviour should be criminal: for example, killing or assaulting others, infringing their property rights or inflicting sexual contact on them without their consent. How should we translate our agreement into legislation? Should we create a single offence for each of these behaviours or multiple offences, as current law tends to do? If the latter, should we adopt the law’s current distinctions: between murder and manslaughter, theft and fraud, rape and sexual assault? Or, if not, what other distinctions should we draw?

These questions embody the problem of *offence differentiation* in criminal law. To date, they have received only one serious answer: the *fair labelling principle*. This principle states, roughly, that distinctions among offences should reflect distinctions in the nature and seriousness of the wrongdoing that they criminalise. In this article, I have two aims: to show that fair labelling should not be the sole or main principle governing offence differentiation; and, in so doing, to develop a broader normative account of these decisions.

These aims are worth pursuing for several reasons. First, the fair labelling principle has been hugely influential, across many different areas of the criminal law. Its most frequent applications are to the law of homicide^1^ and the law governing complicity and modes of responsibility, in both domestic^2^ and international criminal law.^3^ But it is also often invoked in other core areas, including sexual offences,^4^ property offences^5^ and non-fatal offences against the person.^6^ Furthermore, it is often invoked in support of new offences, including domestic abuse,^7^ criminal intoxication,^8^ distinct international crimes^9^ and offences involving multiple wrongs or collateral harms.^10^ The logic of fair labelling is also often invoked even when the principle is not mentioned by name. For example, offences aggravated by hostility towards protected characteristics—so-called hate crimes—are often rationalised solely or mainly by the greater harmfulness and culpability of the conduct that they target.^11^

Secondly, the principle has been especially influential in justifying *increased numbers* of criminal offences. In theory, it need not be used in this way: we could use it to argue against the creation of new offences or in favour of simplifying the law, on the ground that similarly wrongful or culpable conduct should be covered by a single offence.^12^ In practice, however, it is nearly always invoked in favour of new offences or of maintaining existing complexity. Although it may originally have been intended as a restrictive principle, it thus now embodies an ‘inflationary logic’,^13^ which makes its influence especially worthy of scrutiny.

Thirdly, despite this influence, the fair labelling principle has almost never been critically scrutinised. Its origins are typically traced to Andrew Ashworth’s work since the 1980s.^14^ But to date, the only serious examination of its normative foundations remains that of James Chalmers and Fiona Leverick.^15^ Chalmers and Leverick’s analysis is rightly seminal. But it is also admittedly ‘preliminary in nature’ and an attempt ‘to sketch out the issues involved’.^16^ Understandably, then, it leaves some key questions unanswered: for example, how we should evaluate particular arguments from fair labelling, or exactly which distinctions among offences the principle requires.

We must begin, then, by reassessing the normative foundations of the fair labelling principle. After some further clarifications (section 2), I examine how the principle might be justified (section 3). Its only plausible justification, I argue, is a duty to ensure that the blame expressed through criminal conviction is allocated justly. Such a duty would require that differently blameworthy conduct be separated into different offences. However, as I then go on to show (section 4), strict adherence to this logic would have absurd results. Specifically, it would require a highly differentiated criminal law, consisting of a large array of complex, overlapping and narrowly defined offences. The demands of fair labelling are not plausibly absolute, I suggest, and other factors must be relevant to offence differentiation decisions.

In the second half of the article, I turn to examine these other factors. I identify two powerful factors that tell against a highly differentiated criminal law, and thus against the demands of fair labelling (section 5). First, such a criminal law would create problems of inefficiency, inconsistency and unpredictability. And second, it would exacerbate the problems associated with charge bargaining: most importantly, and ironically, the risks of unjust blaming. Finally, in section 6, I briefly highlight some further factors that are relevant to offence differentiation. These might tell either in favour of or against the demands of fair labelling, depending on the circumstances. In short, a complete normative account of offence differentiation must attend to various factors. The fair labelling principle, I conclude, should not be our only or main guide.

## 2. Clarifying the Fair Labelling Principle

Despite its influence, the fair labelling principle remains unclear in some key respects. We must therefore begin by clarifying these, before considering how it might be justified.

First, we must distinguish two issues to which the fair labelling principle is relevant: offence *differentiation* and offence *naming*. The former concerns how we should divide criminal conduct among different offences: for example, whether we should have distinct offences of taking others’ property and obtaining it by deception. The latter concerns what the resulting offences should be called: whether we should name these offences ‘theft’ and ‘fraud’ or something else.^17^ Our focus here is on differentiation. But in some contexts in which the fair labelling principle is often invoked, the main issue is naming. The main issue with convicting secondary parties of principal offences, for example, is the inaccuracy of the resulting labels. What, then, is the relationship between these two issues, from a fair labelling perspective?

The answer is that fair labelling at the level of differentiation is a precondition for fair labelling at the level of naming. Ultimately, the principle requires that our offence labels reflect distinctions in the nature and seriousness of offenders’ wrongdoing. To achieve this, however, we must also create offences that reflect these distinctions, to which these labels can then attach. Given this relationship, problems of naming also have a reliable solution. We first ensure that fair labelling is respected in differentiation: that the relevant distinctions are reflected in distinctions among offences. We then give the resulting offences *descriptively accurate* names: names that describe accurately the conduct that constitutes the offence.^18^ Although our interest is in differentiation, fair labelling in this context can thus lead easily to fair labelling in naming.

There is a complication here, however. Sometimes, we might want to use a particular offence name, which might then give us reason to differentiate offences in a particular way. The obvious examples are traditional, non-descriptive offence names like ‘murder’ and ‘rape’. We might want to retain these, instead of replacing them with more descriptive names like ‘intentional killing’ or ‘non-consensual penile penetration’.^19^ Such proposals, however, are not necessarily compatible with the fair labelling principle, for their purpose is often not to reflect accurately the blameworthiness of the offender’s conduct, but rather to harness the symbolic or emotional power of the traditional labels.^20^ Thus, retaining the label ‘rape’ might lead us to differentiate penile penetration from other penetrative sexual assaults even if they are equally blameworthy.^21^ Symbolic power is better seen as a distinct reason for differentiation, which may conflict with the demands of fair labelling.

A second question is: exactly which distinctions does the principle require us to recognise? Advocates of the principle describe the answer in different ways. They talk, for example, of ‘the degree of the offender’s moral guilt’,^22^ ‘the nature and gravity of the offending’^23^ or ‘the extent of wrongdoing and/or harm involved’.^24^ In practice, these seem to refer mainly to type of conduct, type and degree of harm, and level of culpability—as well as, perhaps, to circumstantial factors that affect seriousness, such as the victim’s characteristics.^25^ As shorthand for all these factors, I will use the term *blameworthiness*. I intend this term to refer to both wrongdoing (that is, the conduct for which a person is to blame) and culpability (the extent to which they are at fault for that conduct). As we will see, this usage reflects the concerns that motivate the principle: to convict someone of an offence is to blame them for committing it, so we should ensure that their blameworthiness is reflected in their conviction.

This leads us to a third issue: how should we judge whether types of conduct are differently or similarly blameworthy? There are two possibilities. We could consult *social morality*: that is, we could ask which distinctions in blameworthiness are widely accepted in our society. Or we could consult *critical morality*: we could ask which distinctions we *should* accept, on the basis of sound moral argument. Such distinctions are identifiable, to at least some extent, independently of social practice. I will focus on the latter, ‘critical morality’ interpretation of the fair labelling principle, for this is, in reality, the dominant interpretation: most applications of the principle use standards of critical morality; very few are truly committed to a ‘social morality’ view.^26^

Unfortunately, the language used by proponents of the fair labelling principle sometimes obscures this reality. The principle, they sometimes say, requires us to recognise distinctions that are ‘widely felt’^27^ or ‘meaningful … in the public mind’,^28^ or conceptions of wrongs ‘in society as it is today’.^29^ Although such language suggests a social morality interpretation, it is misleading. In all the works just quoted, the authors in fact go on to ground their claims in moral argument rather than social attitudes. Even the few who more clearly adopt a social morality interpretation are not deeply committed to it. Evidence of which distinctions in blameworthiness the public draw is very rarely used in arguments about offence differentiation.^30^ And where it is not used, its absence is not seen as a deficiency.^31^ Moreover, as we shall see later, the strongest reasons for differentiating offences in accordance with social morality differ from those typically given in favour of the fair labelling principle.^32^ Again, social morality is better seen as a distinct basis for differentiation, which may conflict with the principle’s demands.^33^

A final issue for clarification is the strength of the fair labelling principle. This issue matters because, at first glance, fair labelling is not the only factor that seems relevant to offence differentiation decisions. We have already noted two others: conformity with social morality and the retention of traditional, ‘symbolic’ offence names. But as Chalmers and Leverick highlight, there are several more: for example, impact on conviction rates,^34^ reflecting harm done to victims^35^ and the need for the law to be simple and workable.^36^ These other factors might sometimes tell against the demands of fair labelling. For example, perhaps we could increase conviction rates by creating mitigated offences for, say, ‘date rape’ or killings by drivers.^37^ Yet such conduct is presumably just as blameworthy as other instances of rape or unintentional killing. To what extent does the fair labelling principle require conformity when its demands conflict with others in this way?

In practice, the predominant answer is that fair labelling is a *presumptive* principle. Normally, we should comply with it; but occasionally, conflicting reasons might lead us to depart from its demands.^38^ Thus, fair labelling is not an absolute principle: distinctions in blameworthiness need not always be reflected in distinctions among offences.^39^ But nor does it state merely one reason to be weighed against others in decisions about offence differentiation.^40^ Rather, the idea seems to be that these other considerations are mostly not *valid*: law makers should disregard them, at least where they conflict with the demands of fair labelling.^41^ Hence, mitigated offences of the kinds just mentioned are inherently objectionable, even if they would increase conviction rates: ‘it would simply be inappropriate for the law to lend its authority to [such dubious distinctions]’.^42^ This implies that the foundations of the fair labelling principle are non-consequentialist in character: law makers must have *duties* to conform with it, even where the balance of reasons would otherwise favour conflicting options.

In summary: the fair labelling principle states that distinctions among offences should, at least presumptively, reflect distinctions in the blameworthiness of the conduct that they criminalise, as judged by standards of critical morality. A successful justification of the principle must, in turn, satisfy two criteria: it must explain our reasons to reflect distinctions in blameworthiness in distinctions among offences; and it must explain the (presumptively) mandatory nature of these reasons, that is, why we should (normally) exclude alternative ways of differentiating offences from our consideration. The next question is whether any justification of the principle can satisfy these criteria.

## 3. Justifying the Fair Labelling Principle

Chalmers and Leverick offer two justifications for the fair labelling principle.^43^ The first relates to the risks of *unfair* labelling: offence names must not ‘create a false or misleading impression of the nature or magnitude of the offender’s wrongdoing or encourage an inaccurate conclusion to be drawn’.^44^ The second is the communicative value of fair labelling: offence names ‘communicate information about the offender to a number of different bodies … [who] may form opinions or make decisions about the offender that turn on the information received’.^45^ We shall see that, while both suggestions contain grains of truth, they fail to meet the criteria for a successful justification of the principle. However, their shortcomings point us in a more promising direction: the principle ensures that the blame expressed through criminal conviction is allocated justly.

### A. A First False Start: Rights against Wrongful Condemnation and Stigma

Consider first the risks of unfair labelling. One way of explaining this concern is by pointing to a right against wrongful condemnation. Criminal conviction is a form of condemnation: to convict someone of an offence is to blame them for committing it. But this is legitimate only if the offender is actually to blame for the conduct concerned. If not, then they are wronged by being convicted. We can draw an analogy with the right to reputation. I can legitimately make claims that damage your reputation if those claims are true. But if they are false, then I wrong you by making them. Conviction of the non-blameworthy is thus a kind of ‘moral defamation’.^46^ The fair labelling principle avoids this result by ensuring that offenders’ convictions reflect their blameworthiness.

This is not a convincing argument for fair labelling as a principle of offence differentiation. True, as we noted earlier, some problems of fair labelling are problems of wrongful condemnation: offenders are sometimes convicted of offences with inaccurate names. But, as we also saw, a reliable solution to these problems is descriptive accuracy in naming. The right against wrongful condemnation is thus easy to respect: we must simply ensure that we criminalise only blameworthy conduct and that we give the resulting offences accurate names. The fair labelling principle, by contrast, demands more than this: it requires that differently blameworthy conduct be separated into different offences. The right against wrongful condemnation does not, by itself, explain this demand.

A more promising focus is not wrongful condemnation, but what we might call wrongful *stigma*. Again, the analogy with reputation is helpful. One way to damage someone’s reputation is to say things that would reasonably lead others to think worse of them. Another is to say things that *will actually* lead others to think worse of them—even if that is not warranted by what was said.^47^ Similarly, criminal conviction might be wrong because it amounts, in its meaning, to wrongful condemnation. But it might also be wrong because it will lead others to think the offender more blameworthy than they are—even if the conviction, in its meaning, does not overstate their blameworthiness.

To see how this might occur, consider broad offences that vary greatly in their seriousness, such as robbery. Advocates of the fair labelling principle often worry about these offences: they worry that those convicted of, say, snatching someone’s bag will be associated with armed robbers storming a bank.^48^ We could try to fix this through descriptive naming: by replacing ‘robbery’ with, say, ‘theft achieved by force’. This, however, is unlikely to solve the problem completely, for when we think of a category, we tend to jump to our paradigm example: we do not stop to consider the full range of cases that the category includes or what proportion of these cases lies beyond the paradigm.^49^ Even ‘theft achieved by force’, then, may risk that bag snatchers are thought of like gun-toting bank robbers. Avoiding such wrongful stigma can be a good reason for ensuring that distinctions in blameworthiness are reflected in distinctions among offences.

Still, the right against wrongful stigma is also an unconvincing justification for the fair labelling principle. Again, the principle demands more than the right does: it demands that offence differentiation reflect blameworthiness even where alternative options would not risk wrongful stigma. Consider again the examples of mitigated offences for certain sexual assaults or unintentional killings. As I noted earlier, such proposals might be motivated by a desire to increase conviction rates or to reflect social morality. They presumably depart from the demands of fair labelling: the conduct concerned is similarly blameworthy to other conduct of the same type. But by hypothesis, this departure consists in the relevant offenders being stigmatised *less* than they might be. Their rights against wrongful stigma are thus not at issue.^50^

The same seems true of some examples of differentiation that are meant to reflect distinctions in blameworthiness. Take the creation of a distinct offence of domestic abuse, or of offences aggravated by hostility. These offences, their proponents argue, recognise that this conduct is differently blameworthy from that targeted by pre-existing offences: for example, public order offences or offences against the person.^51^ But they were not meant to prevent wrongful stigma—to prevent, say, those convicted of mere assault from being stereotyped as hate criminals or domestic abusers. Indeed, there was presumably no such problem, given that hate crime and domestic abuse have historically been under-acknowledged.^52^ From a fair labelling perspective, differentiation thus seems to be justifiable even where no problem of wrongful stigma exists.

In short, applications of the fair labelling principle do not always serve to avoid wrongful condemnation or stigma. While the right against wrongful stigma might generally tell against broadly defined offences, it does not require that distinctions among offences always reflect distinctions in blameworthiness. There is a more general lesson here: calls for fair labelling do not always benefit offenders. A duty to comply with the principle, of the kind that we are seeking, is thus unlikely to be grounded in their interests alone.

### B. A Second False Start: The Communicative Value of Fair Labelling

‘[T]he most convincing argument for fair labelling,’ Chalmers and Leverick say, ‘is that offence names communicate information about the offender to a number of different bodies.’^53^ Convictions, and thus criminal records, state only of what the offender was convicted. For audiences who must interpret these, offence names are thus a crucial source of information. One important such audience is actors within the criminal justice system: especially sentencing judges assessing previous convictions.^54^ But offence names also communicate to audiences outside the system: most importantly, employers,^55^ and, via the media, the general public.^56^ Chalmers and Leverick say some things about the differing needs of these audiences. However, they do not explain in detail how these needs might justify fair labelling as a principle of differentiation.^57^ Do they have a legitimate interest in fine-grained descriptions of blameworthiness, of the kind that the principle demands?

To answer this question, we can usefully separate two sorts of use that we might have for information about offenders’ conduct. The first is *forward-looking*: we might want to use it as evidence of how they will behave in future. We might be concerned about the risk that they pose, for example, or their suitability for a job. Such uses seem incapable of justifying the fair labelling principle. They might give us some reason to differentiate offences according to the type of conduct involved: for example, if one type of offending generally carries a higher risk of reoffending than another. But there is little reason to suppose that finer distinctions in the blameworthiness of past conduct reflect distinctions in likely future behaviour.^58^ Even if they did so, many other factors influence that likelihood: for example, the offender’s sex, age, and past and current life circumstances.^59^ And the principle does not require that these be reflected in offence differentiation and naming. Overall, it is doubtful that individual convictions generally—much less the specific distinctions in blameworthiness that they might report—provide especially relevant evidence for forward-looking judgments.^60^

The second type of use for information about offenders’ past conduct is *backward-looking*. The information might be relevant, that is, to how we should treat offenders in light of their conduct, regardless of their likely future behaviour. At first glance, this seems a stronger justification for the fair labelling principle. Information about blameworthiness is, by definition, relevant to how we should treat people in light of their conduct. However, we need to clarify what kind of treatment we are talking about here. On the one hand, we might be talking about mere blaming judgments or the adoption of blaming attitudes towards the offender. As it is sometimes put: we might be concerned with ‘how [the] offender should be regarded’.^61^ Ideally, we might want beliefs about offenders’ conduct to reflect a complete picture of their blameworthiness. But such detail in our beliefs is not obviously of overriding value. At least, it is not obviously so important that it typically outweighs the competing concerns that are at stake in offence differentiation decisions: for example, conviction rates, or simplicity and workability in the law.

On the other hand, perhaps we are talking not just about blaming judgments and attitudes, but about blaming actions. Perhaps we are talking, for example, about the shunning of offenders, or their exclusion from certain spheres. Such actions certainly seem more important than mere judgments or attitudes. However, it is questionable whether the state should be trying to enable them. If criminal conviction and punishment are themselves meant to be proportionate to the offender’s blameworthiness, then what space for further legitimate retribution remains? This is not to say that private individuals and companies must interact on unchanged terms with offenders who have served their sentences; rather, it is to question whether the state should effectively be ‘outsourcing’ such extra condemnation and punishment to them. If this is the main justification for the fair labelling principle, then it seems at least contentious.^62^

The same objections do not apply to backward-looking uses of information about offending by criminal justice actors. Again, the most important example is the use of previous convictions as an aggravating factor in sentencing. Clearly, this is not a trivial matter, and it concerns state rather than private punishment. And while we might question whether previous convictions should be used in this way, this practice is near-universal and deeply embedded.^63^ Still, it faces the same question: do sentencing judges have a legitimate interest in the fine-grained distinctions in blameworthiness with which the fair labelling principle is concerned? Probably not. The precise seriousness of previous convictions is not obviously relevant on any rationale for treating them as an aggravating factor.^64^ Indeed, in some jurisdictions, seriousness is not relevant at all. For example, in English law, what matters is whether the previous offence was of the same broad type as the present one.^65^ The value of fair labelling to criminal justice actors thus also seems a weak justification for the principle.

None of this is to deny that these audiences have an interest in accurate naming. If offenders’ convictions are to be communicated, then it is important that they describe accurately the conduct for which the offender was convicted. The issue is rather what information we have reason accurately to communicate—and hence how we have reason to differentiate offences. It does seem plausible that there are backward-looking reasons for our communications to reflect offenders’ blameworthiness. Yet these reasons cannot easily be derived from the value of these communications to different audiences. This therefore seems an unstable foundation for the fair labelling principle.

### C. A Fresh Start: Duties to Blame Justly

The fair labelling principle, we saw earlier, must be grounded in some kind of duty. Yet, as we can now see, this duty is unlikely to derive from either the interests of offenders or the instrumental value of fair labelling. So where else might it derive from? The only plausible answer, on which I focus in the rest of this article, is that fair labelling is required as a matter of justice.

Duties of justice have several characteristic features, all of which the fair labelling principle shares.^66^ They concern allocation: in this case, the allocation of blame. They demand that relevantly like cases be treated alike, and that relevantly different cases be treated differently. This explains the thought with which I began this section: that it is wrong to blame the blameless. But it also explains features of the principle that we have so far struggled to explain: that we ought to blame the blameworthy, and that our blaming should reflect how blameworthy they are and for what. Duties of justice also take a non-consequentialist and non-instrumentalist form: to at least some extent, they require conformity regardless of the costs and benefits. Again, this explains an otherwise-confusing feature of the principle: its insensitivity to the instrumental value, or lack thereof, of communications of blameworthiness.

The idea that principles of justice should govern blaming—as well as praising, punishing, rewarding and similar actions—is deeply plausible. To see this clearly, it may help to consider an analogy from outside the law. Suppose you are grading a batch of student essays. What principles should you follow? The obvious answer is that your grades should reflect the quality of the essays. This means, of course, that essays should fail only if they are unsatisfactory. But it also means that they should pass if they are satisfactory, and that the precise grade awarded should reflect the precise quality of the work. Additionally, you should comply with these principles irrespective, to at least some extent, of the consequences of doing so. A lower grade might help to motivate a complacent student, while a higher one might be a welcome boost for someone who is having a hard time. But such concerns are invalid: if you act on them, you act unjustly. Your only guide should be the grade that the essays deserve.

For our purposes, we need not provide a decisive argument for duties to blame justly. The point is that they are the only plausible foundation for the fair labelling principle. We can thus assume that they exist for argument’s sake.^67^ Still, the idea that expressions of blame should be sensitive to blameworthiness is difficult to resist. Arguably, indeed, we can understand blaming only by understanding why it should reflect blameworthiness. To judge someone blameworthy is to judge them in light of their conduct: that is, to judge that their conduct reflects badly on them. And to express blame is simply to communicate such a judgment. It is natural, then, to regard fit with blameworthiness as at least a necessary condition for the legitimacy of such judgments and their expression.^68^ If we are going to impose criminal convictions on offenders, these should plausibly reflect what they are to blame for and to what extent.

From these points, it is only a short step to recognising fair labelling as a principle of offence differentiation. Since conviction expresses blame, it should be subject to the principles of justice that apply to blaming in general. And since conviction is always of an offence, distinctions among convictions require distinctions among offences. Duties to blame justly must thus be fulfilled through offence differentiation. To continue the analogy: if grading must reflect the quality of the work, then so must the marking scheme.

In criminal justice, just blaming is also better delivered through offence differentiation than by other means. In particular, we could try to reflect blameworthiness through sentencing: by treating certain factors as aggravating or mitigating. On this approach, however, the means of communicating blameworthiness is sentence severity. And this has limited potential to convey clearly the nature and seriousness of the offender’s wrongdoing, compared to offence names.^69^ We could remedy this to some extent by requiring judges to address the relevant factor in their sentencing remarks. But even then, the factor will not be part of the conduct for which defendants are charged, tried and convicted. Nor will it feature on offenders’ criminal records.^70^ Blaming through sentencing is thus less clear, and less prominent in the criminal process, than blaming through conviction. From the perspective of justice, the ideal would thus be to recognise distinctions in blameworthiness in the substantive law.

Duties to blame justly, then, are a plausible foundation for the fair labelling principle. But even assuming that they exist, two questions arise. Do these duties plausibly exclude all other considerations from offence differentiation decisions? And if not—if some other considerations remain valid—do they always or normally outweigh these other considerations? I will argue that other considerations remain valid, and that fair labelling reasons do not plausibly have overriding weight. The fair labelling principle cannot, therefore, be the sole or main principle governing offence differentiation.

## 4. The Strength of Fair Labelling Reasons

Reasons to blame justly, and to enable this through offence differentiation, clearly have some intuitive force. Imagine that our criminal law were only minimally differentiated: that it contained only a single offence,^71^ or a small number of broadly defined offences, such as assault, unlawful killing or infringing property rights.^72^ A plausible objection to such a criminal law would be its failure to respect the demands of fair labelling: significant distinctions in blameworthiness would not be recognised in convictions or criminal records.^73^ It is less clear, however, how far the same objection applies to our current criminal law. The distinctions in blameworthiness that currently go unrecognised are much more fine-grained. Must the substantive law reflect all such distinctions? Or must it reflect only some—implying that the demands of fair labelling are not maximally strong?

The latter is by far the more plausible option. To see why, consider again a point that I just noted: sentencing can also reflect blameworthiness, albeit less perfectly than labelling can. In current law, no logic determines whether factors relevant to blameworthiness are reflected in sentencing, the substantive law or both.^74^ For advocates of the fair labelling principle, this is at least presumptively objectionable for, as we just saw, from the perspective of just blaming, distinctions in blameworthiness would ideally be recognised through the substantive law. Once we appreciate how many such distinctions there are, however, this ideal seems absurd. This suggests that other considerations besides duties to blame justly must be valid in offence differentiation decisions.

Recall that blameworthiness consists in both wrongdoing (the conduct for which we are to blame) and culpability (the extent to which we are at fault). Taking wrongdoing first, the most frequently recognised distinctions in the substantive law concern the type of harm caused or risked. Thus, we have distinct families of offence relating to life, person, property, sexual autonomy and so on. Other harm-related distinctions, by contrast, are more often recognised only through sentencing.^75^ Consider degree of harm caused. In English law, offences against the person are graded based on degree of injury, while property offences are completely insensitive to the value of the property destroyed or taken. Or consider victim characteristics, such as vulnerability, or whether the victim was a public servant undertaking their duties. These are relevant to labelling in some contexts,^76^ but are more often relevant to sentencing alone. Still other distinctions are recognised almost exclusively through sentencing: for example, collateral impact on bystanders or the community.

Another kind of distinction in wrongdoing is *how* harm is caused or risked. Some such distinctions are recognised within some families of offence: most obviously, sexual offences and property offences.^77^ But others that are generally relevant at sentencing are typically unrecognised in the substantive law. There is no general scheme of aggravated offences recognising, for example, use of a weapon, or restraint or humiliation of the victim. Similar things can be said of circumstantial elements. Some offences are differentiated mainly by context of commission, such as injury, killing and endangerment on the roads. But other contextual elements are mainly a sentencing matter: for example, commission in a family or domestic context, or abuse of a position of trust.^78^

The extent to which current law fails to satisfy the demands of fair labelling becomes even clearer when we turn to culpability. The substantive law of course distinguishes different forms of *mens rea*, such as intention and recklessness. But while some families of offence are differentiated by *mens rea—*most obviously homicide—many others are not. Distinctions *within mens rea* are also significant to culpability, but are recognised only at sentencing. For example, intended crimes might be spontaneous or premeditated. And reckless crimes vary hugely in their culpability: they include both low risks and near-certainties of harm, and apply regardless of whether the offender cared about their materialisation.^79^

Culpability is also affected by factors beyond *mens rea*. These are recognised almost entirely through sentencing. The obvious example is motive, which has traditionally been irrelevant to substantive criminal liability. A well-known recent exception to this rule is hate crime legislation. But it is unclear why this should be the only exception. Why not also create aggravated offences for conduct motivated by, say, greed or cruelty? Likewise, partial excuses are almost entirely irrelevant to substantive criminal liability.^80^ Yet they can significantly affect culpability. Immaturity, mental disorders, learning disabilities and involvement through coercion or exploitation are all general mitigating factors. While few fair labelling advocates call for the creation of mitigated offences to differentiate these cases,^81^ the logic underlying the principle implies that this should be the ideal.

In short, the logic of fair labelling requires, at least ideally, a far more differentiated criminal law than we currently have. It requires that the substantive law reflect distinctions in type, degree, manner and circumstances of causing or risking harm, as well as distinctions among and within forms of culpability. Every possible configuration of these factors would, as an ideal matter, be reflected in a distinct offence. We should thus create offences of, say, intentional and premeditated breaking of a vulnerable victim’s nose, motivated by revenge; or of recklessly creating a low risk of destroying property worth under £100, mitigated by mental disorder. This result is, I assume, absurd: no reasonable law reformer endorses or would endorse it.^82^ Yet it seems inevitable if, in decisions about offence differentiation, our duties to blame justly exclude or typically override any countervailing concerns.

How might advocates of the fair labelling principle resist this conclusion? The obvious response is that only significant distinctions in blameworthiness need be reflected in the substantive law. This response is not obviously available to those who would advocate an absolute version of the principle. But, as we saw earlier, fair labelling is normally treated as an ideal that may be compromised. On this view, our duties to blame justly are stronger when the stakes are higher: when the distinctions concerned are more significant, or when the offending is more serious.^83^ They are weaker, by contrast, for less significant distinctions among less serious offences.^84^ Again, we might compare sentencing here. Distinctions in blameworthiness are best reflected through conviction; but they can still be reflected to some extent in aggravation, mitigation and judges’ sentencing remarks. This non-ideal option may be good enough when the stakes are lower.

This response is fair, but it does not contradict the point being made here. Its argument is that distinctions in blameworthiness need be recognised only if they are significant. But on the most obvious interpretation, a ‘significant’ distinction is simply one that we have *strong enough* reason to recognise. So, this concession makes sense only if there are valid countervailing factors that we must balance against our reasons for just blaming. This creates a dilemma for advocates of a presumptive fair labelling principle. Either the balance typically favours fair labelling, in which case the principle seems correct, but the absurd results highlighted above will tend to follow; or it does not, in which case we avoid absurdity, but the principle seems incorrect. Instead, fair labelling seems to be just one factor among others that should be considered in offence differentiation decisions. To know which scenario to assume, we must examine the potential countervailing factors, as the following sections will do.

Alternatively, perhaps only certain kinds of distinction count as ‘significant’ for the purposes of the principle. As some put it: it requires us to recognise *qualitative* moral distinctions.^85^ However, if these include qualitative distinctions in culpability, then much of the absurdity identified above remains. Consider distinctions among forms of *mens rea*, between conduct that is and is not partially excused, or that is and is not performed with certain bad motives. All are qualitative distinctions that, if recognised, would lead to a far more differentiated criminal law than we currently have.

A more likely argument is that the principle requires us to recognise only qualitative distinctions in wrongness. Indeed, its advocates sometimes talk as though their task is to divide distinct wrongs into distinct offences. One unanswered question here is how we are to distinguish one wrong from another.^86^ For argument’s sake, let us assume that the distinctions currently recognised through sentencing and the substantive law are a good guide. If so, then this limitation also seems ineffective. Again, many of the distinctions highlighted above are qualitative: for example, those relating to circumstances, victim characteristics and mode of commission. Even differentiation by type of harm could be finer than it is now.^87^ To reflect even qualitative distinctions in wrongness, we would thus need a significantly more differentiated criminal law.

More importantly, to focus on wrongdoing alone would be arbitrary. The fair labelling principle is grounded in duties to blame justly; but wrongdoing is blameworthy only if, and to the extent that, it is culpable. Blaming that is insensitive to culpability is therefore unjust. For this reason, differentiation by wrongdoing alone would have implausible results. It would rule out existing examples of differentiation by culpability: most obviously, in the law of homicide, which is considered a paradigmatic example of fair labelling.^88^ Focusing on qualitative distinctions in wrongdoing also thus seems an unattractive way of rescuing the fair labelling principle from absurdity.

In short, if we were faithfully to apply an absolute or presumptive fair labelling principle, the result would be an absurdly highly differentiated criminal law. This implies that our duties to blame justly cannot be maximally strong: other, countervailing considerations must also be valid. Like any *reductio ad absurdum*, however, this argument is not conclusive. For advocates of the principle could yet embrace the result: they could argue for maximum differentiation despite its implausibility. We must therefore examine the potential countervailing factors and assess their strength.

## 5. Reasons against Greater Differentiation

Many factors besides duties to blame justly seem relevant to decisions about offence differentiation. Some of these factors tend to favour a less differentiated criminal law. This section examines two powerful such factors. Neither is due solely to differentiation: both reflect more general problems with the criminal process, especially as it operates in adversarial systems. These problems tend, however, to be exacerbated by adherence to the demands of fair labelling.

### A. Rule of Law Values

First, the demands of fair labelling will tend to conflict with rule-of-law values. Adherence to the principle, we have seen, will tend to produce a large and complex array of narrowly defined offences. This result threatens ideals of clarity and intelligibility in the law. Even advocates of the principle, in its presumptive form, acknowledge that this concern is valid: they worry that complexity risks inefficient decision making by criminal justice actors.^89^ They rarely acknowledge, by contrast, the related risks of inconsistency in that decision making and consequent unpredictability. From a rule-of-law perspective, these are arguably the greater risks: they limit the extent to which the law both governs official conduct and guides that of citizens.^90^

To see how, we must first note a further effect of offence differentiation. So far, we have focused on only one of these: that it determines the available range of convictions and labels. Also important, however, is that it determines the available range of *charges*. In adversarial systems, choices among charges determine the focus of criminal trials: they determine the allegations to which defendants must plead, and that must be proved if they are to be convicted. And decisions about how to charge defendants, and/or which charges to continue with, are a matter for prosecutors’ discretion.^91^ By increasing the range of available charges, we thus increase the potential influence of this discretion on the focus and outcome of criminal trials.

For this reason, a more complex range of narrower offences will tend to create problems at several stages of the criminal process. It will make it harder for prosecutors, and/or the police, to choose which offence(s) to charge. For cases that go to trial, it will create difficulties of proof: a larger number of more specific elements will need to be established. Alternatively, the same difficulties will confront factfinders, especially juries, if they are required to choose among verdicts. One valid concern at each of these stages is ‘unmeritorious technical argument’: time-consuming debate over unimportant minutiae, which may risk the failure of prosecutions.^92^ But another is the risk of inconsistency. The complexity of these choices, combined with their discretionary nature, risk making the ‘law in action’ a product mainly of official decision-making, rather than of the ‘law in the books’.^93^

Number and complexity of offences will also tend to make the law less predictable for citizens. This point is obvious on one level: criminal law is typically thought to create conduct rules for citizens, so it is generally better that offences be few and simply defined.^94^ Understanding the law gets harder—even, perhaps, with the benefit of expert advice—as offences become more numerous and complex.^95^ More importantly, unpredictability also results from the inconsistency just highlighted. Understandably, citizens will be more interested in the law in action than in the law in the books: they will want to know whether a given behaviour will actually attract the authorities’ attention, and what might happen to them if it does. But these outcomes are difficult to predict to the extent that they turn on discretionary choice.^96^ In these ways, the ‘law professor’s dream’ of fair labelling risks becoming ‘the citizen’s nightmare’.^97^

These risks are less serious if the relevant factor is dealt with at sentencing, rather than through the substantive law. Sentencing law is not typically thought to create conduct rules for citizens, so there is not the same pressure for it to be intelligible to them.^98^ Nor are sentencing decisions complicated in the same way by large arrays of aggravating and mitigating factors. Unlike decisions whether to charge or convict, they are not binary: they are choices among sentences within a continuous range.^99^ Also, the factors that sentencers should consider are not completely determined by the offence of which the offender was convicted. ‘Technicalities’ are thus relatively unlikely to disrupt the sentencing process. Of course, sentencing also involves discretion, and can thus be inconsistent. But by addressing inconsistency in charging and conviction, we address at least one source of this inconsistency.^100^ From a rule-of-law perspective, then, we have stronger reason to avoid fine-grained assessments of blameworthiness in the substantive law than we do at the sentencing stage.

A few caveats are required here. First, clarity and intelligibility do not always or necessarily demand fewer, less specific offences. Again, we might imagine a criminal law containing only a few broadly defined crimes. The likely vagueness of its provisions would also undermine rule-of-law values: especially in regulatory contexts, where detailed schemes of specific offences will often serve clarity.^101^ Likewise, avoiding complexity is not the only way of ensuring clarity and intelligibility. We might also achieve this by, for example, ensuring that the law reflects distinctions that are significant within social morality.^102^ The point here is simply that these values generally provide reasons to avoid complexity. So, they will generally count against the demands of fair labelling, given the large numbers of narrow and complex offences that these tend to favour.

Secondly, some kinds of distinction among offences risk greater inefficiency and inconsistency than others do. Distinctions based on vague criteria, such as normative standards, are an obvious example. Hence, we might want to avoid more extensive schemes of partial defences that rely on reasonableness standards,^103^ or new offences for accomplices that are graded by degree of causal contribution.^104^ Other bases for differentiation, by contrast, might create lesser risks. Consider aggravated versions of existing offences, where the aggravating elements are matters of objective fact that are relatively easy to prove.^105^ Such offences still increase complexity, and they do not change the discretionary nature of charging decisions. But they create fewer problems of proof, and of choice between charges and verdicts. In such cases, our reasons against fulfilling the demands of fair labelling are relatively weak.

Finally, clarity and intelligibility are not absolute values: they must be balanced against justice in blaming. The need for such a balance is familiar from the history of criminal law reform: ideals of clarity, coherence and simplicity have often clashed with the tendency towards specific offences for behaviours that create distinct concern.^106^ The point here is that this tension is genuine, and there is no single correct way of balancing these values. While some advocates of the fair labelling principle concede this point,^107^ others appear not to—even some who advocate a presumptive version of the principle claim, for example, that fair labelling should always take precedence in relation to more serious crimes.^108^ As we can now see, this is too simplistic. The ideals of both just blaming and compliance with rule-of-law values are threatened to different degrees by different ways of differentiating offences. The balance must thus be struck explicitly, on a case-by-case basis.

### B. Overlapping Charges and the Risks of Charge Bargaining

A second factor that will tend to favour a less differentiated criminal law—and thus to tell against the demands of fair labelling—is the risks associated with overlapping charges. In particular, a more differentiated criminal law enables more *charge bargaining*: settlements of cases whereby defendants plead guilty to one charge in exchange for prosecutors dropping another. One distinctive feature of adversarial criminal justice systems, as I noted above, is that charging decisions are a matter for prosecutors’ discretion. Another is that, if defendants plead guilty, then courts need not investigate their guilt independently. Taken together, these features allow the outcomes of criminal cases to be determined by bargaining between prosecution and defence.^109^ The risk of a more differentiated criminal law is that it gives more bargaining chips to prosecutors: they can use threats of more serious charges to incentivise defendants to plead guilty to less serious ones.^110^

As with other ways of incentivising guilty pleas, the costs of charge bargaining arguably outweigh its benefits. Its main benefit is that it promotes efficiency: guilty defendants are convicted without the expense of a trial or the associated risk of acquittal. By contrast, it has at least three significant costs. First, it increases the risk that guilty defendants will be convicted of a less serious offence—and thus receive a less appropriate label—than the evidence warrants. Second, it incentivises defendants to waive their right to put the prosecution to proof. Finally, and most importantly, it incentivises innocent defendants to plead guilty.^111^ Since we normally weigh convictions of the innocent more heavily than we do acquittals of the guilty, it is questionable whether efficiency justifies these risks.

Although the scale of these risks is difficult to measure, they are certainly real and probably significant. Since charging decisions are discretionary, and pleas relatively unchecked, the law leaves ample scope for bargains that do not reflect the merits of the case.^112^ Such outcomes are also likely in practice, given the incentives on both sides. For prosecutors, the certainty of some conviction will often be preferable to the risk of acquittal. And for defendants, the certainty of conviction for a less serious offence will often be preferable to the risk of conviction for a more serious one.^113^ Against this background, it is unsurprising that significant minorities of those who plead guilty maintain their innocence, and at least sometimes credibly so.^114^

This general picture is supported by research on areas of the law that are already significantly differentiated. Charges often move downwards in these areas, to an extent that cannot be explained by the strength of the evidence alone. In offences against the person cases, for example, only a minority of the most serious charges avoid downward movement.^115^ And of those rape charges that result in conviction, as many as half may be for a lesser offence than rape.^116^ In some areas, the picture is more complex. Take offences aggravated by hostility, for which non-aggravated or ‘basic’ forms of the offences present an obvious opportunity for charge bargaining. Some suggest that cultural change has now made it unacceptable for prosecutors to take this opportunity.^117^ However, this has partly just displaced the problem: initial overcharging may now go uncorrected, and defendants may end up (unlawfully) convicted of both basic and aggravated offences.^118^ Overall, the evidence suggests that at least some efficiency-driven charge bargaining takes place even in this context.^119^ If we increase differentiation in other areas of the law, there is no reason to expect different results.

One might respond that, while the risks of incentivising guilty pleas are real, they are not attributable to offence differentiation—for charge bargaining is not the only means of encouraging guilty pleas. The most interesting such means for our purposes is *fact bargaining*: defendants pleading guilty in exchange for prosecutors’ agreement to a more favourable version of the facts, which might lead to a lighter sentence. Again, it is for prosecutors to present a factual basis for sentencing. And if the defendant does not contest this, the court need not review the facts fully.^120^ Prosecutors can thus use aggravating factors as bargaining chips in the same way that they use more serious charges.

This response makes a valid point: the risks of fact bargaining are similar in nature to those of charge bargaining. However, the scale of the latter is probably greater. For one thing, the threat of a more serious charge is different in kind to that of a less favourable factual basis for sentencing. It is a threat of a more serious conviction and label—and thus of potentially greater stigma.^121^ For another, the offence of which one is convicted determines at least the starting point for sentencing. The effects of charge bargaining on sentencing outcomes can thus be significant. Indeed, research suggests that they are especially significant where criminal laws are highly differentiated, and where sentencers have limited discretion: for example, in those US states that use ‘sentencing grids’. In such systems, sentencing outcomes are a product mainly of charging decisions, and charge bargaining is thus an especially powerful tool.^122^

Even where sentencers have greater discretion, however, charges can influence potential sentence in ways that facts cannot. Under English guidelines, for example, aggravating and mitigating factors are allowed a relatively large and unconstrained influence on sentencing outcomes.^123^ Yet sentencers are still constrained by category ranges for the offence of which the offender was convicted—and the differences between these ranges can be substantial. For example, the least serious cases of rape and assault by penetration have starting points of five and two years’ imprisonment respectively. The equivalent starting points for intentionally causing and simply inflicting grievous bodily harm are four years’ imprisonment and a community order.^124^ While charge bargaining is not the only means of incentivising guilty pleas, then, it is an especially powerful one.

For advocates of the fair labelling principle, these concerns seem both inescapably valid and difficult to balance. On the one hand, adherence to the principle increases the potential for just blaming. It ensures that available offence labels more fully reflect distinctions in blameworthiness; and by enabling charge bargaining, it increases the probability that the blameworthy are convicted of at least something.^125^ On the other hand, the pressure to bargain downwards means that, ironically, new labels will not be applied in many cases where they should be. It also means an increased risk of wrongful conviction—and thus of unfair labelling. Furthermore, we might question the extent to which efficiency is a relevant reason, since it is achieved by inducing defendants to waive their procedural rights. In short, we must weigh perhaps modest increases in just blaming against increased risks of substantive and procedural injustice. At the very least, we should avoid assuming that the former always or normally outweigh the latter.

## 6. Some Further Relevant Factors

Some factors that are relevant to offence differentiation, then, will tend to tell against the demands of fair labelling. Others, by contrast, will tend to strengthen them, albeit in a limited range of cases: for example, avoiding wrongful stigma and reflecting harm done to victims. More often, however, a factor might tell either in favour of or against fair labelling, depending on the circumstances. I have already noted some such factors: for example, reasons to retain symbolic offence names and to communicate distinctions in reoffending risk. In the space that remains, I cannot examine such factors comprehensively. Instead, I will briefly highlight three more, to illustrate their variety and potential importance.

First, if an issue is relevant to conviction, then in more serious cases it will be adjudicated by juries rather than judges. If an issue would be better adjudicated by juries, we thus have reason to make it relevant to conviction.^126^ Two powerful arguments for the use of juries are that they may be better fact-finders than single judges and that it is good to involve lay people directly in governance.^127^ These arguments suggest that, generally, more important issues are better left to juries: both accuracy and lay involvement are more valuable when greater blame and stigma are at stake. They also suggest that some specific types of issue are better left to juries. Most obviously, evaluative questions, such as the reasonableness of the defendant’s conduct or beliefs, may be better answered by groups of lay people. Jury responsibility will thus sometimes add weight to arguments from fair labelling.

Other issues, by contrast, might be less suitable for jury decision. We should avoid leaving juries to make judgments that they find especially difficult: for example, judgments of the degree of probability that the defendant’s actions would cause a given harm.^128^ We should also avoid leaving them judgments that might be undermined by their biases and entrenched opinions. These include, for example, judgments of aggravation or mitigation with which they are likely to disagree.^129^ They also include the application of at least some vague evaluative standards—balancing the reasons just mentioned to leave such judgments to them.^130^ Of course, such worries might also apply to judicial decision making. But since, unlike juries, judges are ‘repeat players’, at least some of these worries might be addressed through experience and training.^131^ Jury responsibility might also thus provide reasons against differentiating offences as the fair labelling principle demands.

Second, offence differentiation is sometimes argued to be a means to broader social change. To see how this might work, recall once again how differentiation through the substantive law is more powerful than differentiation through sentencing: the differentiating factor is reflected in convictions and criminal records, and must be proved or admitted at trial. This more visibly distinct treatment may encourage both the public and criminal justice actors to take the conduct concerned more seriously. This may, in turn, create a virtuous circle: more reports and investigations may lead to more convictions, which may encourage more reports and investigations. Eventually, this may even lead to the creation or strengthening of social norms against the conduct concerned. Differentiation decisions might thus help to reduce the incidence of that conduct, albeit indirectly.

Arguments of this kind are most often made in relation to conduct that, historically, the criminal justice system has failed to take seriously enough. While they often coincide with arguments from fair labelling, they do not always or necessarily do so. Typically, they are arguments for distinct offences: for example, of hate crime^132^ or domestic abuse.^133^ But they might also be arguments against this. One might argue against the creation of degrees of rape, for example, on the basis that all non-consensual sex should be taken equally seriously.^134^ Such arguments should be approached with caution. In all the contexts just mentioned, social attitudes have been slow to change, and substantive law reform has not brought about that change directly or by itself. Still, there is no good argument for supposing that criminal law cannot help to change social morality, and some reason to believe that it has done so in the past.^135^ These might thus be valid arguments for differentiation, at least to the extent that it is part of a wider, realistic strategy.

Thirdly, however, a desire to harness criminal law’s expressive power might also lead us to differentiate offences in other ways. In particular, it might require us to conform with social morality, rather than trying to change it. This argument has been made most forcefully by Paul Robinson. Drawing on a range of different types of evidence, Robinson argues that people’s willingness both to comply with criminal law and to co-operate in its enforcement depend on its accordance with social morality. This argument applies to both particular laws and the criminal justice system in general. Hence, criminal law can sometimes secure compliance and co-operation where its demands differ from those of social morality; but only if the two generally match up.^136^ This requires not only punishing the socially blameworthy and acquitting the socially blameless, but also matching conviction to degree of social blameworthiness.^137^ In other words: this is an argument for a version of the fair labelling principle based on social rather than critical morality.^138^

Once again, this principle is not absolute. Other values might outweigh it—including those I examined earlier, such as rule-of-law values and procedural fairness.^139^ And again, we might seek to change social morality by changing the law, including through offence differentiation. If Robinson is correct, however, then the law’s ability to do this depends on its generally following social morality. While departures in the name of critical morality may seem justifiable individually, a general policy of allowing them will be costly. In Robinson’s phrase: criminal law only gets so many ‘credibility chips’.^140^ These should not be spent indiscriminately, as the fair labelling principle tends to warrant.

## 7. Conclusions

The fair labelling principle states that distinctions among offences should reflect distinctions in the blameworthiness of the conduct that they criminalise. Despite this principle’s widespread influence, this article has argued that it should not be the sole or main principle governing offence differentiation decisions. Its only plausible foundation is a duty to ensure that the blaming inherent in criminal conviction is allocated justly. But this duty cannot be absolute, and its demands must be weighed against other valid and potentially countervailing considerations. These include reasons to avoid creating large numbers of narrow, complex and overlapping crimes. They also include other reasons deriving from the effects of offence differentiation on charge, trial and conviction. A complete normative account of offence differentiation must accommodate these reasons, and thus extend beyond the fair labelling principle.

## Footnotes

1 For a very much non-exhaustive list, see eg A Ashworth and B Mitchell (eds), *Rethinking English Homicide Law* (OUP 2000); Law Commission, *Murder, Manslaughter and Infanticide* (Law Com No 304, 2006); W Wilson, ‘The Structure of Criminal Homicide’ [2006] Crim LR 471; W Wilson, ‘What’s Wrong with Murder?’ (2007) 1 Criminal Law and Philosophy 157; CMV Clarkson and S Cunningham (eds), *Criminal Liability for Non-Aggressive Death* (Routledge 2008); G Maher, ‘“The Most Heinous of All Crimes”: Reflections on the Structure of Homicide in Scots Law’ in J Chalmers, F Leverick and L Farmer (eds), *Essays in Criminal Law in Honour of Sir Gerald Gordon* (Edinburgh UP 2010); J Horder, *Homicide and the Politics of Law Reform* (OUP 2012); H Keating and J Bridgeman, ‘Compassionate Killings: The Case for a Partial Defence’ (2012) 75 MLR 697; A Cornford, ‘The Architecture of Homicide’ (2014) 34 OJLS 819; M Gibson and A Reed, ‘Reforming English Homicide Law: Fair Labelling Questions and Comparative Answers’ in A Reed and M Bohlander (eds), *Homicide in Criminal Law: a Research Companion* (Routledge 2019); Scottish Law Commission, *Discussion Paper on the Mental Element in Homicide* (Discussion Paper No 172, 2021).

2 See eg R Sullivan, ‘First Degree Murder and Complicity—Conditions for Parity of Culpability Between Principal and Accomplice’ (2007) 1 Criminal Law and Philosophy 271; D Husak, ‘Abetting a Crime’ (2014) 33 Law and Philosophy 41; DJ Baker, *Reinterpreting Criminal Complicity and Inchoate Participation Offences* (Routledge 2016). The wider critical literature on criminal complicity is also typically occupied with fair labelling concerns, without necessarily referring to them as such. For a good example, see D Yeager, ‘Helping, Doing and the Grammar of Complicity’ (1996) 15 Criminal Justice Ethics 25.

3 See eg D Guilfoyle, ‘Responsibility for Collective Atrocities: Fair Labelling and Approaches to Commission in International Criminal Law’ (2011) 64 CLP 255; JG Stewart, ‘The End of “Modes of Liability” for International Crimes’ (2012) 25 LJIL 165; M Jackson, ‘The Attribution of Responsibility and Modes of Liability in International Criminal Law’ (2016) 29 LJIL 879.

4 See eg Home Office, *Setting the Boundaries: Reforming the Law on Sex Offences* (2000); H Power, ‘Towards a Redefinition of the Mens Rea of Rape’ (2003) 23 OJLS 379; Scottish Law Commission, *Report on Rape and Other Sexual Offences* (Scot Law Com No 209, 2007); M Gibson, ‘Deceptive Sexual Relations: A Theory of Criminal Liability’ (2020) 40 OJLS 82; SP Green, *Criminalizing Sex: A Unified Liberal Theory* (OUP 2020).

5 See eg CMV Clarkson, ‘Theft and Fair Labelling’ (1993) 56 MLR 554; S Shute and J Horder, ‘Thieving and Deceiving: What Is the Difference?’ (1993) 56 MLR 548; AL Bogg and J Stanton-Ife, ‘Protecting the Vulnerable: Legality, Harm and Theft’ (2003) 23 LS 402; AP Simester and GR Sullivan, ‘On the Nature and Rationale of Property Offences’ in RA Duff and SP Green (eds), *Defining Crimes: Essays on the Special Part of the Criminal Law* (OUP 2005); SP Green, *Thirteen Ways to Steal a Bicycle: Theft Law in the Information Age* (Harvard UP 2012); G Betts, ‘Robbery and the Principle of Fair Labelling’ (2019) 83 JCL 205.

6 See eg J Gardner, ‘Rationality and the Rule of Law in Offences Against the Person’ (1994) 53 CLJ 502; J Horder, ‘Rethinking Non-Fatal Offences Against the Person’ (1994) 14 OJLS 335; Law Commission, *Reform of Offences Against the Person* (Law Com No 361, 2015); M Gibson, ‘Getting Their “Act” Together? Implementing Statutory Reform of Offences Against the Person’ [2016] Crim LR 597.

7 V Tadros, ‘The Distinctiveness of Domestic Abuse: A Freedom-Based Account’ in Duff and Green (n 5). See also several of the contributions to M McMahon and P McGorrery (eds), *Criminalising Coercive Control: Family Violence and the Criminal Law* (Springer 2020).

8 R Williams, ‘Voluntary Intoxication—a Lost Cause?’ (2013) 129 LQR 264.

9 HM Zawati, *Fair Labelling and the Dilemma of Prosecuting Gender-Based Crimes at the International Criminal Tribunals* (OUP 2014); T de Souza Dias, ‘Recharacterisation of Crimes and the Principle of Fair Labelling in International Criminal Law’ (2018) 18 International Criminal Law Review 788.

10 B Mitchell, ‘Multiple Wrongdoing and Offence Structure: A Plea for Consistency and Fair Labelling’ (2001) 64 MLR 393.

11 See eg MA Walters, ‘Conceptualizing “Hostility” for Hate Crime Law’ (2014) 34 OJLS 47: invoking these as sufficient reasons for distinct offences, while acknowledging that such offences may have limited instrumental value. Similarly, others regard it as decisive against such offences that hate crime is *not* necessarily more harmful or culpable: see eg HM Hurd and MS Moore, ‘Punishing Hatred and Prejudice’ (2004) 56 Stan L Rev 1081.

12 Arguments of this kind are very rare in the literature. Perhaps the most significant is the argument against the graded schemes of sexual assault offences that have been enacted in some jurisdictions: that these have unjustifiably downgraded some forms of rape. See eg J Temkin, *Rape and the Legal Process* (2nd edn, OUP 2002) ch 3; Home Office (n 4) 16.

13 L Farmer, *Making the Modern Criminal Law: Criminalization and Civil Order* (OUP 2016) 114. As we will see later (section 4), this point is not merely true in practice: the logic of the fair labelling principle actually is inflationary.

14 For what is normally taken to be the original statement of the principle, see A Ashworth, ‘The Elasticity of Mens Rea’ in C Tapper (ed), *Crime, Proof and Punishment: Essays in Memory of Sir Rupert Cross* (Butterworth 1981).

15 J Chalmers and F Leverick, ‘Fair Labelling in Criminal Law’ (2008) 71 MLR 217.

16 ibid 246.

17 The distinction is Chalmers and Leverick’s. In their metaphor: differentiation is the question of which box we put offenders in; naming is the question of what we write on the box: ibid 222. I prefer ‘naming’ to Chalmers and Leverick’s label of ‘description’: as we will see, offences names might or might not be descriptive in character.

18 Although Chalmers and Leverick do not put it this way, this seems consistent with the spirit of their analysis. They prize descriptive accuracy and are correspondingly sceptical about ‘emotive’ offence names, although they do not reject them outright: ibid 241–2.

19 See eg Law Commission, *Murder, Manslaughter and Infanticide* (n 1) 22; Temkin (n 12) 177–8.

20 In which case, accurate naming must instead be achieved by ensuring that the definition of the offence matches public understanding of the label concerned: Cornford (n 1) 834–8.

21 Compare J Gardner and S Shute, ‘The Wrongness of Rape’ in J Gardner, *Offences and Defences: Selected Essays in the Philosophy of Criminal Law* (OUP 2007) 21–5; S Cowan, ‘All Change or Business as Usual? Reforming the Law of Rape in Scotland’ in C McGlynn and VE Munro (eds), *Rethinking Rape Law: International and Comparative Perspectives* (Routledge 2010) 159–61.

22 G Williams, ‘Convictions and Fair Labelling’ (1983) 42 CLJ 85.

23 Mitchell (n 10) 394.

24 Clarkson (n 5) 555.

25 Chalmers and Leverick (n 15) 242–3.

26 This is generally true of the sources cited at nn 1–10 above. The vast majority of these arguments are clearly grounded in moral claims made, as it were, from the armchair, rather than in claims about social attitudes. For a more general defence of a ‘social morality’ fair labelling principle—the only one of which I am aware—see V Tadros, ‘Fair Labelling and Social Solidarity’ in L Zedner and JV Roberts (eds), *Principles and Values in Criminal Law and Criminal Justice: Essays in Honour of Andrew Ashworth* (OUP 2012).

27 Mitchell (n 10) 393.

28 Simester and Sullivan (n 5) 186.

29 Horder, ‘Rethinking Non-Fatal Offences’ (n 6) 335.

30 For a rare example of a serious attempt to integrate such evidence into an argument about offence differentiation, see Green, *Thirteen Ways to Steal a Bicycle* (n 5). Even Green, however, ultimately wants to show that critical morality vindicates the relevant social morality.

31 For the only real acknowledgement of these problems that I know of—focusing, in particular, on the questions that they raise for multicultural and multilingual societies—see K Moshikaro, ‘The Moral and Legal Foundations of Fair-Labelling in Our Criminal Law’ (2018) 135 SALJ 262, 265, 281–3. Simply put, for Moshikaro, what is important is not the fact that a community uses given labels or moral concepts, but that these pick out behaviours whose distinct wrongness is rationally explicable. In the terms that I am using here: social morality matters only because, and to the extent that, it tracks critical morality.

32 See section 6 below.

33 Again, this is consistent with Chalmers and Leverick’s analysis. While they recognise reasons for the law to avoid being ‘utterly out of step with public opinion’, they suggest that critical morality should prevail where the two conflict: Chalmers and Leverick (n 15) 241.

34 ibid 240, referring to this as ‘pragmatism’.

35 ibid 236–8.

36 ibid 239–40.

37 For critical discussion of the latter example, see S Cunningham, ‘Vehicular Homicide: Need for a Special Offence?’ in Clarkson and Cunningham (n 1). See n 12 above on gradations of rape.

38 This was certainly Ashworth’s view. In his words: fair labelling is a ‘principle’ and not ‘an absolute injunction’: A Ashworth, *Principles of Criminal Law* (1st edn, OUP 1991) 72. Again, this fairly describes the majority of academic engagements with the principle cited so far. (Perhaps unsurprisingly, law reform bodies tend to treat it more as one valid consideration to be balanced against others.)

39 Although there are at least apparent exceptions to this rule: sometimes, distinctions in blameworthiness are treated as *decisive* reasons for creating new offences. For an illustrative recent example, see Gibson (n 4), arguing for a new scheme of offences covering deceptive sexual relations that would be distinct from, but parallel to, the current sexual offences. While accepting that both types of offence would involve the same type and degree of harm, Gibson argues that they would involve distinctive ways of wrongfully bringing about that harm, and treats this as a decisive reason for differentiating them.

40 For more on such distinctions among principles of criminalisation, see eg V Tadros, *Wrongs and Crimes* (OUP 2016) 93–101; RA Duff, *The Realm of Criminal Law* (OUP 2018) 249–53. One question here is what distinguishes presumptive principles from *pro tanto* or ‘one reason among others’-type principles. For our purposes, the difference need only be one of degree: that is, of how frequently the relevant reason is defeated by others.

41 The one apparent exception is the need for simplicity and workability. Chalmers and Leverick, along with other advocates of a presumptive version of the principle, seem to regard this as a valid countervailing reason: see further section 5A below.

42 Chalmers and Leverick (n 15) 240.

43 They also convincingly reject several other putative justifications, namely: that it helps to control sentencing discretion (ibid 224–5), that it helps to educate offenders and prevent reoffending (229–30) and that it ensures fairness to victims (236–7).

44 ibid 228.

45 ibid 238.

46 ibid 228. See also AP Simester and A von Hirsch, *Crimes, Harms, and Wrongs: On the Principles of Criminalisation* (Hart Publishing 2011) 19–20.

47 For a helpful defence of this view, and exploration of its implications, see A Slavny, ‘The Normative Foundations of Defamatory Meaning’ (2018) 37 Law and Philosophy 523.

48 See eg A Ashworth, ‘Robbery Re-Assessed’ [2002] Crim LR 851; Betts (n 5).

49 This tendency reflects cognitive biases that are well known to psychologists, such as stereotyping and base-rate neglect. See RF Pohl (ed), *Cognitive Illusions: A Handbook on Fallacies and Biases in Thinking, Judgement and Memory* (2nd edn, Psychology Press 2016) especially chs 2 and 11.

50 One might reply that the wrongful stigma here accrues to those who are convicted of non-mitigated offences that cover similarly blameworthy conduct. However, whether this is true is a contingent matter: it depends on whether the existence of the mitigated offence causes those convicted of the non-mitigated offence to be seen as more blameworthy than they are. By contrast, from a fair labelling perspective, such offences are objectionable regardless of whether they have such an effect. So again, the avoidance of wrongful stigma cannot entirely explain the principle’s demands.

51 See nn 7 and 11 above.

52 As we will see later, the historic failure to take such conduct seriously enough is another reason that has been offered in favour of these offences: see section 6 below.

53 Chalmers and Leverick (n 15) 238.

54 ibid 231–3.

55 ibid 234–5.

56 ibid 226–9.

57 In fact, Chalmers and Leverick’s overall conclusion is that differentiation is more important for audiences within the criminal justice system, whereas accurate naming is more important for those outside it: ibid 246. As should already be apparent, however, this cannot be the whole story—for what gets communicated through accurate naming depends on differentiation. The key question, then, is whether the different audiences have interests in receiving different information—and hence whether this gives us reason to differentiate offences in different ways.

58 Research suggests that, generally speaking, people do not ‘specialise’ in types of offence, and that number and frequency of previous convictions are much more powerful predictors of reoffending than is their type. See CJ Sullivan and SM Gann, ‘Crime Specialization, Progression, and Sequencing’ in G Bruinsma and D Weisburd (eds), *Encyclopedia of Criminology and Criminal Justice* (Springer 2018) for a summary of the literature. Nor, notably, do employers themselves generally take these distinctions seriously: approaches vary, but most will discriminate against candidates simply because they have criminal records. See T Thomas and B Hebenton, ‘Dilemmas and Consequences of Prior Criminal Record: A Criminological Perspective from England and Wales’ (2013) 26 Criminal Justice Studies 228, 235–6.

59 Research on desistance from crime makes this point clear. See eg J Shapland and A Bottoms, ‘Desistance from Crime and Implications for Offender Rehabilitation’ in A Liebling, S Maruna and L McAra (eds), *The Oxford Handbook of Criminology* (6th edn, OUP 2017).

60 You might respond that this is an argument against any blanket losses of rights or liberties attaching to criminal records alone—as opposed to, say, individualised risk assessment of offenders. I think this is a bullet that we should be prepared to bite: see Z Hoskins, *Beyond Punishment? A Normative Account of the Collateral Legal Consequences of Conviction* (OUP 2019) 173–5.

61 Chalmers and Leverick (n 15) 226.

62 For further development of a similar argument in a different context, see Hoskins (n 60) 137–42. Hoskins argues that, beyond their sentences, offenders have a claim to full restoration of their status, at least as far as their relationship with the state is concerned.

63 For a recent comparative analysis, including discussion of possible rationales for the practice, see J Roberts and S Harrendorf, ‘Criminal History Enhancements at Sentencing’ in K Ambos and others (eds), *Core Concepts in Criminal Law and Criminal Justice*, vol I (CUP 2020).

64 ibid 291–2.

65 For example, whether it was also an offence of violence: A Ashworth, *Sentencing and Criminal Justice* (6th edn, CUP 2015) 219–20. Chalmers and Leverick likewise identify the issue here as whether ‘previous convictions are in the same broad general area as the offence for which the offender is being sentenced’. They add only that it may be useful to have ‘information about the context in which an offence took place, such as whether an assault was an incident of domestic violence’: Chalmers and Leverick (n 15) 232. Neither claim provides strong support for the fair labelling principle.

66 See D Miller, ‘Justice’ in EN Zalta (ed), *The Stanford Encyclopedia of Philosophy* (2017) s 1 <https://plato.stanford.edu/entries/justice>.

67 Of course, we can also imagine arguments against such duties. The most troubling challenges are those that afflict retributive forms of justice in general: how could the expression of blame be intrinsically good? And can we explain this other than by appeal to intuition? For a good overview of the terrain, see A Walen, ‘Retributive Justice’ in EN Zalta (ed), *The Stanford Encyclopedia of Philosophy* (2020) <https://plato.stanford.edu/entries/justice-retributive/>.

68 For an engaging recent defence of this view, see J Gardner, ‘Why Blame?’ in I Solanke (ed), *On Crime, Society, and Responsibility in the Work of Nicola Lacey* (OUP 2021).

69 Chalmers and Leverick (n 15) 223.

70 At least, not by default. We could require that certain aggravations be noted on criminal records, as has sometimes been proposed: see eg Law Commission, *Hate Crime: Should the Current Offences Be Extended?* (Law Com No 348, 2014) 65–74. In that case, the gap between conviction and sentencing is narrowed, at least as far as blaming is concerned.

71 For example, of endangering others’ legally protected interests: L Alexander and KK Ferzan, *Crime and Culpability: A Theory of Criminal Law* (CUP 2009) ch 8.

72 For perhaps the most famous proposal of this kind, see L Blom-Cooper and T Morris, *With Malice Aforethought: A Study of the Crime and Punishment for Homicide* (Hart Publishing 2004).

73 Chalmers and Leverick (n 15) 223.

74 The distinction is not, as one might initially think, one of importance. Aggravation and mitigation often overwhelm the offence committed in determinations of offence seriousness. See generally Ashworth, *Sentencing and Criminal Justice* (n 65) ch 5.

75 Most of the examples of factors relevant to sentencing mentioned in this and the next few paragraphs are drawn from the general list in the Sentencing Council’s *General Guideline: Overarching Principles* (2019) <www.sentencingcouncil.org.uk/overarching-guides/crown-court/item/general-guideline-overarching-principles/>. Many further aggravating and mitigating factors that are relevant to specific offences also go unreflected in the substantive law.

76 The obvious example is the aggravated offence of assaulting an emergency worker: Assaults on Emergency Workers (Offences) Act 2018, s 1. Distinct offences for specific classes of victims are, however, increasingly fertile ground for enthusiastic legislators: see eg Protection of Retail Workers (Retail and Age-Restricted Goods and Services) (Scotland) Act 2021.

77 Distinctions of this kind among property offences are a *locus classicus* of the fair labelling literature: see n 5 above. A more recent example is the much-trumpeted new offence of strangulation or suffocation, which targets a type of conduct already criminalised by multiple overlapping offences, when committed in a particular way: Serious Crime Act 2015, s 75A, inserted by the Domestic Abuse Act 2021.

78 ‘Mainly’ because there are some exceptions to the rule: eg the offences of fraud by abuse of position (Fraud Act 2006, s 4) and of controlling or coercive behaviour, which is limited in its scope to intimate and family relationships (Serious Crime Act 2015, s 76).

79 For critical reflection, see RA Duff, ‘Two Models of Criminal Fault’ (2019) 13 Criminal Law and Philosophy 643.

80 Partial defences to murder are, of course, the notable exception. This is a rare context in which recognition through offence labelling has been explicitly compared to recognition through sentencing alone: see eg Law Commission, *Murder, Manslaughter and Infanticide* (n 1) 48–50.

81 For a rare exception, see J Horder, *Excusing Crime* (OUP 2004) 143–6. A cynic might wonder whether this lack of interest in mitigation tells us something about the forces that sustain fair labelling advocacy.

82 Sensible reformers might advocate radical changes to criminal law and procedure, such that recognition through conviction no longer requires the creation of a distinct offence. Some argue, for example, that criminal law should have separate rules governing citizens’ conduct and assessments of culpability and grading, and that assessment of these matters should be separated procedurally: see, most famously, PH Robinson, *Structure and Function in Criminal Law* (OUP 1997). In that case, it would not seem so absurd at least to record convictions in such a way. The argument here assumes, however, that such reforms will not be forthcoming.

83 Horder, ‘Rethinking Non-Fatal Offences’ (n 6) 342–3.

84 For example, some argue that it is not so urgent to distinguish thefts by the precise value of the property stolen: Simester and Sullivan (n 5) 187.

85 See eg Horder, ‘Rethinking Non-Fatal Offences’ (n 6) 341–2; Mitchell (n 10) 399.

86 I leave this question aside here. However, it might well reveal further difficulties for advocates of the fair labelling principle. For some reasons to believe this, see L Farmer, ‘Criminal Wrongs in Historical Perspective’ in RA Duff and others (eds), *The Boundaries of the Criminal Law* (OUP 2010).

87 See generally Horder, ‘Rethinking Non-Fatal Offences’ (n 6) for a good example of a proposal of this kind.

88 See n 1 above.

89 This is the only factor that Chalmers and Leverick unambiguously conclude should be balanced against the demands of fair labelling: see Chalmers and Leverick (n 15) 239–40. The same goes for Ashworth: see Ashworth, *Principles of Criminal Law* (n 38) 73–4.

90 See generally J Raz, *The Authority of Law: Essays on Law and Morality* (OUP 1979) ch 11.

91 For a helpful recent analysis of these issues, see JI Turner and T Weigend, ‘Negotiated Case Dispositions in England, Germany and the United States’ in Ambos and others (n 63) 390–407. As noted there, there are also important differences among adversarial systems on these points: for example, charging decisions in England are subject to review and to duties laid down in guidelines in ways that they are not in the United States.

92 As noted above, this is the risk that preoccupies advocates of the fair labelling principle. See eg Williams (n 22) 93; Horder, ‘Rethinking Non-Fatal Offences’ (n 6) 340–2; Mitchell (n 10) 410–11; Simester and Sullivan (n 5) 187.

93 The risk is especially high in our modern world of broadly drawn criminal laws, which are capable of catching the ordinary behaviour of large proportions of the population. See WJ Stuntz, ‘The Pathological Politics of Criminal Law’ (2001) 100 Mich L Rev 505, 519–23; D Husak, *Overcriminalization: The Limits of the Criminal Law* (OUP 2007) 20–6.

94 This point should not be overstated. A complex scheme of specific homicide offences, for example, will not necessarily disrupt the communication of the basic conduct rule ‘do not kill’. The point is just that, the more complex a scheme of offences becomes, the harder it gets to derive such conduct rules from it. Again, a radical solution would be to separate conduct rules from assessments of their violation: see n 82 above.

95 There are few better demonstrations of this point than Chalmers and Leverick’s own empirical research in another context. See eg J Chalmers and F Leverick, ‘Quantifying Criminalization’ in RA Duff and others (eds), *Criminalization: the Political Morality of the Criminal Law* (OUP 2014); J Chalmers and F Leverick, ‘Criminal Law in the Shadows: Creating Offences in Delegated Legislation’ (2018) 38 LS 221.

96 Husak, *Overcriminalization* (n 93) 27–31.

97 Husak, ‘Abetting a Crime’ (n 2) 64.

98 This is not to say, of course, that sentencing law can legitimately be unintelligible to citizens; rather, intelligibility is not a concern in the same way or to the same degree that it is in the substantive law.

99 This is, of course, a simplification. There are also different types of sentence, and some that are available only upon conviction for certain offences. Mostly, however, courts can choose among a range of possible sentences for any given offence.

100 The extent to which this is true will vary between jurisdictions, depending on the extent to which conviction influences sentence. See further section 5B below.

101 J Horder, ‘Bureaucratic “Criminal” Law: Too Much of a Bad Thing?’ in Duff and others, *Criminalization* (n 95) 109–13.

102 Gardner (n 6) 512–15.

103 N Lacey, ‘Partial Defences to Homicide: Questions of Power and Principle in Imperfect and Less Imperfect Worlds …’ in Ashworth and Mitchell (n 1) 125–8.

104 Compare J Dressler, ‘Reforming Complicity Law: Trivial Assistance as a Lesser Offence?’ (2008) 5 Ohio State Journal of Criminal Law 427.

105 See eg Mitchell (n 10) 410–11, defending the creation of distinct offences for incidents involving multiple instances of already-criminal conduct.

106 See eg Horder, *Homicide and the Politics of Law Reform* (n 1) ch 2, on homicide offences; Farmer (n 13) chs 7 and 8, on property offences and offences against the person.

107 For more explicit acknowledgements, see eg Ashworth and Mitchell (n 1) 16; Simester and Sullivan (n 5) 187; Husak, ‘Abetting a Crime’ (n 2) 64.

108 See eg Horder, ‘Rethinking Non-Fatal Offences’ (n 6) 341–3.

109 Again, the analysis in Turner and Weigend (n 91) is helpful here.

110 Husak, *Overcriminalization* (n 93) 36–8.

111 These costs and benefits are well rehearsed in the extensive literature on plea bargaining and sentence discounts for guilty pleas. For good recent overviews, see M Vogel, ‘Plea Bargaining under the Common Law’ in DK Brown, JI Turner and B Weisser (eds), *The Oxford Handbook of Criminal Process* (OUP 2019); J Gormley, R McPherson and C Tata, *Sentence Discounting: Sentencing and Plea Decision-Making: Literature Review* (Scottish Sentencing Council 2020).

112 Again, there are differences among adversarial jurisdictions in just how much scope is left. For example, according to the English *Code for Crown Prosecutors* (CPS 2018), prosecutors should accept guilty pleas only if this will enable ‘a sentence that matches the seriousness of the offending’ (para 9.2), and they ‘must never accept a guilty plea just because it is convenient’ (para 9.3). However, as we will see in this and the following paragraph, there is little incentive for them to adhere strictly to this guidance, and ample evidence that they do not do so in practice.

113 For further discussion and references, see A Sanders, R Young and M Burton, *Criminal Justice* (4th edn, OUP 2010) 463–81; L Campbell, A Ashworth and M Redmayne, *The Criminal Process* (5th edn, OUP 2019) 322–5.

114 Sanders, Young and Burton (n 113) 486–9.

115 D Moxon and C Hedderman, ‘Mode of Trial Decisions and Sentencing Differences Between Courts’ (1994) 33 Howard Journal of Criminal Justice 97, 100; E Genders, ‘Reform of the Offences Against the Person Act: Lessons from the Law in Action’ [1999] Crim LR 689, 690–1; R Henham, ‘Further Evidence on the Significance of Plea in the Crown Court’ (2002) 41 Howard Journal of Criminal Justice 151, 153. Genders in particular suggests that, while the strength of the evidence supporting the more serious charge is an important factor in this movement, it cannot entirely explain it.

116 J Harris and S Grace, *A Question of Evidence? Investigating and Prosecuting Rape in the 1990s* (Home Office 1999) 30–2; SJ Lea, U Lanvers and S Shaw, ‘Attrition in Rape Cases: Developing a Profile and Identifying Relevant Factors’ (2003) 43 BJC 583, 592–3. A meta-study covering five anglophone countries found similar average results: K Daly and B Bouhours, ‘Rape and Attrition in the Legal Process: A Comparative Analysis of Five Countries’ (2010) 39 Crime and Justice 565, 568. cf L Kelly, J Lovett and L Regan, *A Gap or a Chasm? Attrition in Reported Rape Cases* (Home Office 2005) 72–4, finding that, while half of the convictions in their study were due to guilty pleas, only a third of those were to lesser offences. The relevant lesser offences are mostly other sexual assaults or child sexual offences, although not exclusively so.

117 A Owusu-Bempah, MA Walters and S Wiedlitzka, ‘Racially and Religiously Aggravated Offences: “God’s Gift to Defence”?’ [2019] Crim LR 463, 470–1.

118 ibid 471–4.

119 See eg E Burney and G Rose, *Racist Offences—How Is the Law Working?* (Home Office Research Study 244, 2002) 78–9; HMCPSI, *A Follow-Up Review of CPS Casework with a Minority Ethnic Dimension* (2004) 26–7; Law Commission, *Hate Crime* (n 70) 120–1.

120 Once again, these points are broadly true of adversarial systems, although there are differences among them. For a helpful recent review of the English position, see M Wasik and A Ashworth, ‘Issues in Sentencing Procedure’ [2020] Crim LR 397. Importantly, defendants have some incentive to avoid disputing the prosecution statement of facts after pleading guilty, as they risk a cut in their sentence discount.

121 This threat has proved powerful in some of the contexts mentioned above. For example, the stigma of terms like ‘racist’ and ‘rapist’ plays a role in bargaining in those contexts. See eg Temkin (n 12) 152–3; Owusu-Bempah, Walters and Wiedlitzka (n 117) 475.

122 RF Wright and RL Engen, ‘The Effects of Depth and Distance in a Criminal Code on Charging, Sentencing, and Prosecutor Power’ (2006) 84 NCL Rev 1935.

123 See n 74 above.

124 The starting points are drawn from the Sentencing Council’s current Crown Court guidelines <www.sentencingcouncil.org.uk/crown-court/>. Note that, in both pairs, the more serious offence is entirely included within the less serious one; in theory, then, the difference in starting point might be due solely to the choice of charge.

125 Of course, from a fair labelling perspective, a conviction that does not fully reflect the offender’s blameworthiness is less than ideal. The point here is just that it might coherently be preferred to no conviction at all.

126 Of course, in reality, only a small minority of cases are adjudicated by juries. This suggests that we ought to take the effects of offence differentiation on pretrial bargaining, highlighted earlier, more seriously than we take these effects on adjudication.

127 See generally M Redmayne, ‘Theorising Jury Reform’ in A Duff and others (eds), *The Trial on Trial: Volume Two: Judgment and Calling to Account* (Hart Publishing 2006).

128 J Horder and D Hughes, ‘Comparative Issues in the Law of Homicide’ in J Horder (ed), *Homicide Law in Comparative Perspective* (Hart Publishing 2007) 15–17.

129 For example, in the hate crime context, juries appear reluctant to accept the law’s insistence that demonstrations of hostility be treated the same as motivation by hostility: Owusu-Bempah, Walters and Wiedlitzka (n 117) 479–81.

130 The classic example is the use of such standards in partial defences to murder. It is at least arguable that provocation would not have proved so problematic if it had been relevant only to sentencing, rather than the substantive law. See Horder, *Homicide and the Politics of Law Reform* (n 1) 229–35.

131 See generally VP Hans and RK Helm, ‘Procedural Roles: Professional Judges, Lay Judges, and Lay Jurors’ in DK Brown, JI Turner and B Weisser (eds), *The Oxford Handbook of Criminal Process* (OUP 2019).

132 Walters (n 11) 54–6.

133 Tadros, ‘The Distinctiveness of Domestic Abuse’ (n 7) 133–4.

134 V Tadros, ‘Rape Without Consent’ (2006) 26 OJLS 515, 540. See also n 12 above.

135 See L Green, ‘Should Law Improve Morality?’ (2013) 7 Criminal Law and Philosophy 473, 479–86.

136 See generally PH Robinson, *Intuitions of Justice and the Utility of Desert* (OUP 2013) especially chs 8 and 9.

137 ibid 208–9.

138 For a similar argument, see generally Tadros, ‘Fair Labelling’ (n 26).

139 Robinson (n 136) 196.

140 ibid 198.

